# Symptoms of Depression, Anxiety, and Stress and Prevalence of Major Depression and Its Predictors in Female University Students

**DOI:** 10.3390/ijerph18115845

**Published:** 2021-05-29

**Authors:** Vanessa Blanco, Mar Salmerón, Patricia Otero, Fernando L. Vázquez

**Affiliations:** 1 Department of Evolutionary and Educational Psychology, University of Santiago de Compostela, 15782 Santiago de Compostela, Spain; 2 Department of Clinical Psychology and Psychobiology, University of Santiago de Compostela, 15782 Santiago de Compostela, Spain; marimar.salmeron@usc.es (M.S.); fernandolino.vazquez@usc.es (F.L.V.); 3 Department of Psychology, University of A Coruña, 15008 A Coruña, Spain; patricia.otero.otero@udc.es

**Keywords:** depression, anxiety, stress, major depression, predictors, university students

## Abstract

Depression, anxiety and stress are increasingly concerning phenomena in our society, with serious consequences on physical and mental health. The repercussions may be particularly devastating in particular population subgroups, such as female university students. The purpose of this study was to determine the levels of depression, anxiety, and stress and the prevalence of depression and associated factors, in Spanish university women. A cross-sectional study was conducted with a random sample of 871 students from the Santiago de Compostela University (mean age 20.7 years, SD = 2.8). Information was collected on sociodemographic and academic characteristics; symptoms of depression, anxiety, and stress; diagnosis of major depression; optimism, resilience, social support, life engagement, and five personality domains, using validated instruments. Of the participants, 18.1%, 22.8% and 13.5% presented with severe/very severe levels of depression, anxiety and stress, respectively. A total of 12.9% had major depression. Higher life engagement was associated with lower risk of depression (OR = 0.92, 95% CI 0.87–0.98), while higher levels of neuroticism (OR = 1.20, 95% CI, 1.12–1.28) and openness to experience (OR = 1.08, 95% CI, 1.02–1.14) were associated with greater risk. These findings reveal an alarming percentage of female university students who experience major depression and severe/very severe stress.

## 1. Introduction

Emerging adulthood is considered the key age group for the onset of various mental health issues [1,2]. At this stage of life, 30–40% of people in high-income countries are pursuing university studies [3,4]. This means that they are subjected to a series of stressors related to academic demands, including a high workload, an extensive curriculum, long class hours, a lack of leisure time, frequent exams, competition with peers, concerns related to academic performance, and a fear of failure, among others [5]. Further compounding those stressors are psychosocial stressors related to independence, such as living away from family, planning one’s future as an adult, pressure to succeed in a competitive job market, and the constant need to make decisions [6]. Combined, these stressors make students a highly significant group for the study of mental health. In fact, 20–30% of university students each year experience mental health problems [7,8]. These problems can have significant negative repercussions, such as impaired social functioning and academic performance, and can lead to dropping out and even suicidal behavior [7,9,10]. The most common mental health problems among students are depression and anxiety, especially for women (e.g., [11]).

Given the special vulnerability of university students to mental health problems, previous studies have used the 21-item Depression Anxiety and Stress Scales (DASS-21 [12]) to evaluate symptoms of depression, anxiety, and stress in university students of both sexes in different countries (e.g., [7,13,14,15,16,17,18,19,20,21]). However, to our knowledge, no study has analyzed the symptoms (and their frequency) of depression, anxiety, and stress specifically in a sample of female university students. This would be highly relevant information at the clinical level for the design of interventions adapted to the needs of this population. In addition, some studies have focused on very specific subgroups of students, such as first-year university students (e.g., [13,15]) or students studying medicine (e.g., [7,16,17]). In Spain, only two studies [18,19] have analyzed this question in the university population. Odriozola-González et al. [18] collected data on mental health symptoms in the context of confinement due to COVID-19 and they included teaching and administrative staff in the study sample without providing disaggregated data for students. Ramón-Arbués et al. [19] studied a sample of 1074 students from three departments at the University of San Jorge in Zaragoza, Spain, and found that 18.4%, 23.6%, and 34.5% presented scores outside the normal range on the subscales for depression, anxiety, and stress, respectively.

Of the most frequent mental disorders in university students, major depression (e.g., [7,8]) is particularly prevalent among women (e.g., [1]). In systematic reviews and meta-analyses of the prevalence of depression in university students of both sexes, percentages ranged from 11.0% for Asian university medical students [22] to 30.6% for university students in various academic fields in different countries [1]. Given that these review studies used different time frames of reference, if we focus specifically on studies that analyzed the current prevalence of major depression, many of them diagnosed depression based on a screening instrument (e.g., [23,24,25]), resulting in diverse rates of prevalence depending on the instrument used and the established cut-off point. Fewer studies have used diagnostic instruments [26,27,28,29,30,31,32]; those prevalence figures range from 0.4% in Chinese students [29] to 10.5% in Brazilian students [28]. Specifically, to our knowledge, there have been only two previous studies examining the current prevalence of major depression of the female university student population using a diagnostic instrument, both conducted at Spanish universities. In the first, Vázquez et al. [31] found a 10.4% prevalence of current major depression in 365 female students. In the second, Vázquez et al. [32] examined a sample of 1043 students and found a 5.3% prevalence of single-episode depression and 3.7% for recurrent episodes, resulting in a combined prevalence of 9%.

A series of studies have analyzed predictors of depression in the university population. Ibrahim et al. [1] indicate that the most commonly studied sociodemographic predictors include age, with inconclusive age-related differences, and family socioeconomic status, with lower rates found in students from higher-income families. This is consistent with Farrer et al. [24], who found higher rates of depression among students who were struggling financially. With respect to other living conditions, Roh et al. [33] found a higher prevalence of depression among college students who lived alone, and El-Gendawy et al. [34] found depression to be more prevalent among those who resided in rural areas. In addition, no relationship between marital status and the prevalence of depression has been identified [35], though several studies have found a higher prevalence of depression in non-heterosexual college students (e.g., [36,37]). To our knowledge, there are no studies that have specifically analyzed the relationship between economic independence and the prevalence of depression in the university population, but one previous study among university women [32] found that economically independent students had higher rates of mental health problems than those who were financially dependent on others.

Some academic predictors have been found to be associated with depression. For example, the highest rates of depression have been found among those who were in their first years of university [1]. However, one review study conducted with university students in Brazil [38] found that students in their final years had a higher prevalence of depression than those in their earlier years. In addition, Ahmed et al. [23] found a higher prevalence of depression among students of social sciences and humanities.

Furthermore, in relation to clinical predictors, various studies have found an inverse correlation between optimism and depressive symptoms (e.g., [39,40]), and low resilience has been associated with a higher risk of depression [40,41]. There is little research on the relationship between life engagement and depression; Liu et al. [42] and Rossi et al. [43] found negative correlations between this psychological resource and depressive symptoms and depression. However, while Rossi et al. [43] found that life engagement was a predictor of depressive symptoms, Liu et al. [42] did not. With regard to social support, Curran et al. [44] found higher rates of depression among persons with lower levels of social support, and Liu et al. [42] found that positive social relationships were predictive of depressive symptomatology in this population.

Finally, in relation to personality variables, various previous studies [45,46,47,48] have established that higher levels of neuroticism predict depression, but the relationship between other personality factors and depression has received less attention. Aldridge and Gore [45] examined the relationship between personality traits and psychological well-being in American college students and found that neuroticism, agreeableness, conscientiousness, and openness to experience, but not extraversion, predicted depressive symptoms. However, of the five major personality traits, only neuroticism has been found to have predictive value [46]. Among the weaknesses of the existing research on predictors of depression in college students is the fact that the evidence for many of these sociodemographic, academic, and clinical variables is limited or inconclusive. In addition, there are no previous studies that have specifically analyzed predictors of depression in Spanish university women.

Given the negative repercussions of the previously addressed mental health problems in the population under study [7,9,10], we must have adequate knowledge of their prevalence and the most frequent symptoms, which will allow to estimate the need for clinical services. The knowledge of the most significant risk and protective factors will make it possible to design interventions tailored to the specific needs of the female university students.

This study examines the symptoms of depression, anxiety, and stress, along with the current prevalence of major depression and its predictors in a random, representative sample of university women from a Spanish university.

## 2. Materials and Methods

### 2.1. Sample

We conducted a cross-sectional study with a random sample of 921 female university students from the University of Santiago de Compostela, located in Galicia, a region in the Northwest of Spain. This region comprises an area of 29,434 km^2^ and has 2,701,743 inhabitants. The recruitment took place between September and November of 2019. A random stratified sampling procedure was used to select the sample from the total of 15,033 female students registered at the university, stratified by major (arts and humanities, sciences, health sciences, social and legal sciences, or engineering and architecture) and level of study (undergraduate or graduate). The participants had to be matriculating in a degree program at the university and be aged 16 years or older to participate in the study. Those who did not provide written informed consent were excluded.

**Figure 1:**
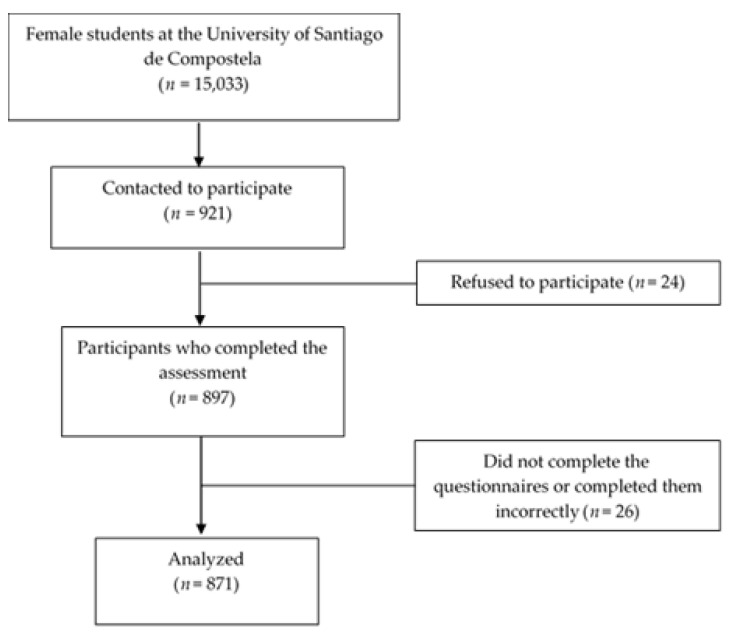
Flowchart of the study participants.

The sample size was calculated using a 10.4% prevalence of depression based on a previous pilot study [30] with a precision ±3%, an alpha error of 5%, and expected sample loss of 15%. It was estimated that a minimum sample of 441 participants would be necessary. The response rate was 97.5%. Initially, 921 participants were contacted, of whom 24 declined to participate and 26 were eliminated due to incomplete data. The final sample was made up of 871 female university students (see Figure 1), with an age range between 16 and 39 years (M = 20.7, SD = 2.8).

To diminish the loss of participants, the suggestions of Hulley, Newman, and Cummings [49] were followed; these included presenting the research in an attractive fashion, avoiding invasive measurement techniques, and using an individual approach to reducing distress. Participation was totally voluntary, and no incentives (academic, financial, or other) were offered. The study was approved by the ethics committee of the University of Santiago de Compostela and followed the principles of the Declaration of Helsinki.

### 2.2. Measures

Clinical experts in mental health made the diagnosis of major depression using an unstructured clinical interview using the criteria of the Diagnostic and Statistical Manual of Mental Disorders, fifth edition (DSM-5 [50]). The following instruments were used:

Sociodemographic and academic variables. An ad hoc questionnaire was developed for this study that gathered information on participants’ age, housing situation during the academic year (lived with other/with friends), financial independence, family area of residence (rural/urban), family income (on a monthly basis), relationship status (single/partnered), sexual orientation (other orientations/heterosexual), academic major (other/social or legal sciences), and level of study (undergraduate/graduate).

Depression Anxiety Stress Scales-21 items (DASS-21 [12]; Spanish version by Bados et al. [51]). The factor structure of the Spanish version is similar to the original scale. It consists of three subscales, containing seven items each, to measure symptoms of depression (items 3, 5, 10, 13, 16, 17, and 21), anxiety (items 2, 4, 7, 9, 15, 19, and 20), and stress (items 1, 6, 8, 11, 12, 14 and 18) during the last week, on a 4-point Likert scale that ranges from 0 (not applicable to me) to 3 (very applicable to me, or applicable most of the time). Two systems were used for correction to facilitate comparability with previous studies. First, the direct scores were obtained for each subscale by adding the scores for all the items in a range from 0 to 21. Second, using the DASS-21 manual’s guidelines as a point of reference, the scores for each subscale were multiplied by two, yielding a range of 0–42. For the depression subscale, scores of 9 or less were estimated to be normal, 10–13 mild, 14–20 moderate, 21–27 severe, and 28 or more extremely severe. For the anxiety subscale, scores of 7 or less were considered normal, 8–9 mild, 10–14 moderate, 15–19 severe, and 20 or more extremely severe. Finally, for the stress subscale, scores of 14 or less were considered normal, 15–18 mild, 19–25 moderate, 26–33 severe, and 34 or more extremely severe. The internal consistency for the Spanish version was 0.84 for depression, 0.70 for anxiety, and 0.82 for and stress.

Life Orientation Test—Revised (LOT-R [52]; Spanish version by Otero et al. [53]). The factor structure of the Spanish version resembles that of the original validation. This is a self-administered 10-item instrument that the participant answers on a 5-point Likert scale, from 0 (I completely disagree) to 4 (I completely agree). Of these, six items measure the dimension of dispositional optimism, while the other four items are filler and serve to make the content of the test less evident. The total score is obtained by adding the scores for items 1, 3, 4, 7, 9 and 10 (having into account that the scores for items 3, 7 and 9 must be previously reversed). The scale range ranges from 0 to 24, with higher scores indicating a greater level of optimism. The internal consistency (Cronbach’s alpha) of the Spanish version was 0.78.

Connor-Davidson Resilience Scale—10-item (CD-RISC 10 [54]; Spanish version by Blanco et al. [55]). The factor structure of the Spanish version resembles that found in the original version. This consists of 10 items that assess resilience on a Likert scale from 0 (not true at all) to 4 (true most of the time). The total score is obtained by adding the direct scores for all the items. Scores range from 0 to 40, with a higher score indicating greater resilience. In the Spanish version, a cut-off of 23 was established, obtaining a sensitivity of 70.0%, a specificity of 68.2%, a positive predictive value of 20.0%, and a negative predictive value of 95.2%. The internal consistency (Cronbach’s alpha) of the Spanish version was 0.86.

Multidimensional Scale of Perceived Social Support (MSPSS [56]). This scale was developed to assess perceived social support in relation to three sources of support: family, friends, and significant others. This study used the Spanish version by Landeta and Calvete [57], whose factor structure resembles that found in the original scale. It consists of 12 items—four for each source of support—which the participant responds to using a 7-point Likert scale ranging from 1 (strongly disagree) to 7 (strongly agree). The total score is obtained by adding the direct scores for all the items, in a range from 12 to 84. The higher the score, the higher the estimated perceived social support. The internal consistency of the Spanish version (evaluated using Cronbach’s alpha) was 0.89 [57].

Life Engagement Test (LET [58]; Spanish version by Lima-Castro et al. [59]). The factorial structure of the Spanish version is similar to the original scale. This is a self-administered scale that assesses life engagement using six items that are evaluated on a 5-point Likert scale that ranges from 1 (totally disagree) to 5 (totally agree). The total score is obtained by adding the direct scores for all the items. The total score ranges from 6 to 30, with higher scores indicating greater life engagement. The internal consistency of the Spanish version (evaluated through Cronbach’s alpha) was 0.81 [59].

Big Five Inventory (BFI-2-S [60]). This evaluates the Big Five personality domains: extraversion, agreeableness, conscientiousness, neuroticism, and openness to experience, using 30 items (six for each domain) with a Likert scale response format ranging from 1 (totally disagree) to 5 (totally agree). The score for the extraversion domain is obtained by adding the scores for items 1R, 6, 11, 16, 21R, 26R; the score for the agreeableness domain by adding the scores for items 2, 7R, 12, 17R, 22, 27R; the score for the conscientiousness subscale by adding the scores for items 3R, 8R, 13, 18, 23, 28R; the score for the neuroticism scale by adding the scores for items 4, 9, 14R, 19R, 24R, 29; and finally, the score for the openness to experience domain by adding the scores for items 5, 10R, 15, 20R, 25, 30R. The items keyed with an R must be reversed. The score for each domain ranges from 6 to 30, with higher scores indicating a greater presence of the trait. In the original validation study, internal consistencies (Cronbach’s alpha) for the different domains ranged from 0.73 to 0.84 in various samples.

### 2.3. Procedure

To systematize the procedure, we developed a protocol that detailed the following parameters of the study: aims, design and framework of the study; the participants (calculation of sample size, sampling procedure, inclusion and exclusion criteria); the study measurement instruments (for both the predictive and outcome variables); biases (non-response, recall, selection); quality control for the method; data administration and analysis; and ethical issues.

Two psychologists outside the research team with 2 years of experience in the assessment and diagnosis of mood disorders were first trained to conduct the evaluation by two experts in the field (a clinical psychologist and a psychiatrist) with more than 25 years of experience. The training comprised four 90-min sessions consisting of seminars and role playing. Subsequently, a pilot study was performed with 20 participants to evaluate the adequacy of the measures, the competence of the assessors, and the feasibility of the research. Information on the sociodemographic and academic characteristics of the participants was collected in a hetero-administered manner. Symptoms of depression, anxiety, and stress; diagnosis of major depression; optimism; resilience; social support; life engagement; and the five personality domains (extraversion, agreeableness, conscientiousness, neuroticism, and openness to experience) was collected in one sitting and took approximately 40 min. After the pilot study, the participants selected for the study were individually contacted and invited to participate. They were informed of the nature, aims, risks and benefits, their confidentiality was guaranteed, and their questions were answered. After providing informed consent, each participant was interviewed in person individually, following the protocol described for the pilot study, in a location convenient for the student.

### 2.4. Data Analysis

The means, standard deviations and ranges were calculated for the continuous variables (scores for the depression, anxiety and stress subscales; optimism; resilience; social support; life engagement; and the extraversion, agreeableness, conscientiousness, neuroticism and openness to experience domains), and frequencies and percentages for the categorical variables (age, housing situation, financial independence, area of residence, family income, relationship status, sexual orientation, academic major, and undergraduate or graduate degree). The differences in the scores for the depression, anxiety and stress subscales according to the sociodemographic and academic characteristics of the participants were analyzed using Student’s *t*-test for independent samples. The differences in the participant´s levels of depression, anxiety and stress as a function of the sociodemographic and academic variables were analyzed using Chi-square tests.

Multivariate logistic regression analyses were performed to analyze the sociodemographic, academic, and clinical variables that may be associated with major depression. First, univariate analyses were performed to independently analyze the relationship between each of the variables and major depression. According to the recommendation of Sperandei [61] for large sample sizes, the variables with a *p* < 0.25 in the univariate analyses were included in the multivariate logistic regression analyses, yielding the corresponding odds ratios (OR) with 95% confidence intervals (95% CI). The analyses were performed using the IBM SPSS statistical package (version 25, IBM Corp., Armonk, NY, USA).

## 3. Results

### 3.1. Sociodemographic, Academic, and Clinical Characteristics

Table 1 shows the participants’ sociodemographic and academic variables. The majority (56.3%) of the participants were ≤20 years old, 51.0% lived with friends, 92.1% were not financially independent, 55.5% had their family residence in urban areas, and 53.0%, had a monthly family income of 2000 Euros or more. Of the interviewed participants, 56.1% did not have a partner, and 73.1% indicated a heterosexual orientation. Furthermore, 62.7% were pursuing a degree in an area other than social/legal sciences and 85.5% were pursuing undergraduate degrees.

Regarding the clinical variables, the mean optimism score was 12.5 (SD = 5.1; range 0–24), resilience was 24.6 (SD = 7.3; range 0–40), social support was 5.9 (SD = 1.0; range 1–7), and life engagement was 23.5 (SD = 4.6; range 7–30). The means and standard deviations were as follows for the five personality characteristics: extraversion (M = 18.4; SD = 4.1; range 7–30); agreeableness (M = 22.9; SD = 3.8; range 10–30); conscientiousness (M = 20.7; SD = 4.7; range 8–30); neuroticism (M = 19.7; SD = 4.8; range 6–30); and openness to experience (M = 22.1; SD = 4.4; range 8–30).

### 3.2. Symptoms of Depression, Anxiety, and Stress and Prevalence of Major Depression

Scores on the depression, anxiety, and stress subscales were 5.6 (SD = 5.3), 4.5 (SD = 4.4) and 6.9 (SD = 4.8), respectively. In relation to the symptoms of depression, 51.9% had normal levels of depression, 13.2% mild, 16.8% moderate, 7.5% severe, and 10.6% very severe. With respect to the anxiety subscale, 52.1% presented with normal anxiety levels, 8.3% mild, 16.8% moderate, 8.7% severe, and 14.1% very severe. Finally, for the stress subscale, 58.6% presented with normal stress levels, 12.6% mild, 15.3% moderate, 9.5% severe, and 4.0% very severe (see Table 2). 12.9% (*n* = 112) of participants had major depression.

Table A1 shows the means and standard deviations for the depression, anxiety and stress subscales according to the sociodemographic and academic variables. Significantly higher depression scores were found in participants ≤ 20 years old (*t* (869) = 3.170, *p* = 0.002), those without a partner (*t* (846.030) = 2.838, *p* = 0.005), those with a sexual orientation other than heterosexual (*t* (376.340) = 3.663, *p* < 0.001), and who were pursuing undergraduate degrees (*t* (189.795) = 3.667, *p* < 0.001). Significantly higher anxiety scores were found for those who were non-heterosexual (*t* (869) = 4.141, *p* < 0.001) and undergraduate students (*t* (869) = 1.978, *p* = 0.048). Finally, higher stress scores were found for those who had a sexual orientation other than heterosexual (*t* (869) = 2.593, *p* = 0.010). No relationship to differences in the scores for the depression, anxiety or stress subscales were found for the other variables.

Table A2 shows the distribution of the levels of depression according to the sociodemographic and academic characteristics; there were differences in the levels of depression between heterosexuals and non-heterosexuals, (χ^2^ (4, *n* = 871) = 13.600, *p* = 0.009). Table A3 exhibits the frequency distribution of the levels of anxiety according to the sociodemographic and academic variables; differences were found in the level of anxiety between heterosexuals and non-heterosexuals, (χ^2^ (4, *n* = 871) = 18.571, *p* = 0.001). Table A4 shows the frequency distribution of the levels of stress according to the sociodemographic and academic characteristics); no significant differences were found in the levels of stress according to any of the sociodemographic or academic variables analyzed. No other differences were found for the levels of depression, anxiety or stress according to the other sociodemographic or academic variables.

Table A5 shows the frequency distribution of the symptoms of depression, anxiety, and stress. For the depression subscale, the symptoms most frequently rated as applicable “most of the time” were feeling downhearted and blue (14.5%) and feeling that one was not worth much as a person (10.9%); the least frequently reported symptoms (“not at all applicable”) were feeling that life was meaningless (70.3%) and having nothing to look forward to (64.3%). For the anxiety subscale, the symptoms most frequently reported as applicable “most of the time” were worrying about panicking or making a fool of oneself (11.4%) and increased heart rate without physical exertion (9.0%); the least frequently reported symptoms were being on the verge of panic (71.9%) and tremors (67.4%). For the stress subscale, the symptoms most frequently reported as applicable “most of the time” were having difficulty relaxing (14.9%) and having a hard time winding down (14.6); the least frequently reported symptoms were being intolerant of obstacles to getting things done (62.0%) and using a lot of nervous energy (48.5%).

### 3.3. Predictors of Major Depression

Variables with a *p* < 0.25 in the univariate logistic regression analyses were family income, relationship status, sexual orientation, optimism, social support, life engagement, extraversion, agreeableness, conscientiousness, neuroticism, and openness to experience.

These variables were included in the multivariate logistic regression analyses (see Table 3). The significant variables in the multivariate analyses were life engagement (OR = 0.92, 95% CI, 0.87–0.98, *p* = 0.009), neuroticism (OR = 1.20, 95% CI, 1.12–1.28, *p* < 0.001), and openness to experience (OR = 1.08, 95% CI, 1.02–1.14, *p* = 0.008).

## 4. Discussion

The aim of this cross-sectional study was to examine the symptoms of depression, anxiety, and stress, along with the current prevalence of major depression and its predictors in a random, representative sample of women from a Spanish university. The participants showed elevated mean scores for the depression, anxiety and stress subscales, and significant percentages of them presented with severe or very severe levels of depression, anxiety and stress, and were suffering from current major depression. Clinical risk and protective factors were identified. These findings have important implications for the fulfillment of the clinical needs of this group of the population.

The sociodemographic profile of the students participating in this study was as follows: women under 20 years of age who lived with friends, not financially independent, mainly resided in an urban area, a monthly family income > than 2000 euros, single, and heterosexual. The majority of the participants were studying a degree in an area other than the social or legal sciences and were pursuing undergraduate degrees. These data are similar to those in the 2020–2021 data and figures report for the Spanish university system published by the Ministry of Universities [3], which found that the majority of students in Spain were women (55.3%) between 18 and 21 years old who were undergraduate students in an area of study other than the social and legal sciences. Furthermore, the data are partially consistent with the work of Ariño et al. [62], who found the majority of the participants lived with their parents (compared to the current study, in which the majority lived with friends), went to school full time (and thus were economically dependent), had mothers with a mean income of less than 1200 euros (60.8%) and fathers with a mean income of less than 2300 euros (64.2%), and were single.

In comparison to previous studies, the scores for the depression subscale were higher than those found by Moutinho et al. [17] for Brazilian medical students, and similar to those found by Cheung et al. [15] for Chinese first-year university students, Kulsoom and Afsar [16] for medical students in a multiethnic context, and Ramón-Arbués et al. [19] for Spanish university students. They were lower than those of Fawzy and Hamed [5] for Egyptian medical students. Scores for the anxiety subscale were higher than those from Moutinho et al. [17], similar to those from Ramón-Arbués et al. [19], and lower than those from Cheung et al. [15], Fawzy and Hamed [5], and Kulsoom and Afsar [16]. Finally, the stress subscale score was lower than those found in all the aforementioned studies [5,15,16,17,19]. Depression scores were higher for those aged 20 or younger, who were single, had a sexual orientation other than heterosexual, and were undergraduate students. These findings are consistent with those from the studies by Ramón-Arbués et al. [19], who found higher depression scores for the students aged less than 21, with no stable partner, and Fawzy and Hamed [5], who found higher depression scores for medical students in their three first academic years, compared to those in their three last academic years. However, Amir-Hamzah et al. [13] and Fawzy and Hamed [5] failed to find a relationship between depression and age, and Cheung et al. [15] and Shamsuddin et al. [20] found higher depression scores for older students. Anxiety scores were higher for those who were non-heterosexual, and those were pursuing an undergraduate degree. These results are consistent with the research by Fawzy and Hamed [5], who found higher anxiety scores for the students in their first three academic years. Stress scores were higher for non-heterosexuals. The finding that depression, anxiety and stress scores were higher in non-heterosexual students is consistent with previous literature (e.g., [63,64]) with first-year college students.

Regarding the participants with severe or very severe levels of depression, anxiety and stress, the data for the depression subscale in the current study is higher than those found by Amir Hamzah et al. [13] and Shamsuddin et al. [20] for Malaysian university students, by Beiter et al. [14] for American university students, and by Ramón-Arbués et al. [19] (both for all students and specifically for women). It was similar to the figures found in the work of ul Haq et al. [21] for university students from Pakistan and lower than those in the work of Fawzy and Hamed [5]. The severe or very severe anxiety levels found in the current study were similar to those from Amir Hamzah et al. [13], higher than those from Beiter et al. [14] and Ramón-Arbués et al. ([19], for both sexes and specifically for women), and lower than those from the studies by Fawzy and Hamed [5], Shamsuddin et al. [20], and ul Haq et al. [21]. Finally, with respect to the stress subscale, the percentage of moderate/severe stress found in this study was higher than that from Amir Hamzah et al. [13], Beiter et al. [14], Ramón-Arbués et al. [19], and Shamsuddin et al. [20]; again, it was lower than the levels found in the works of Fawzy and Hamed [5] and ul Haq et al. [21]. There were differences in the levels of depression and anxiety between heterosexuals and non-heterosexuals. Though, to our knowledge, no previous studies have used the DASS-21 to compare the levels of depression and anxiety among college students as a function of their sexual orientation, this finding is consistent with previous works reporting higher severity of the symptoms in sexual minorities (e.g., [63,64]).

Regarding the predominant symptoms for each subscale, the most frequent depressive symptoms were feeling down-hearted and blue and feeling that one did not have much worth as a person; the least frequent were feeling that life had no meaning and having nothing to look forward to. Although the studies that analyzed symptomatology did not report the most frequent symptoms, two previous investigations [30,31] also found that depressed mood was the most frequent symptom (among students of both sexes, and among female students, respectively, but in both cases specifically for students with depression). However, the results are inconsistent with those from a previous study of Spanish university students with and without depression [65], which found that the most common depressive symptom was hopelessness. In relation to the symptoms of anxiety, the most frequent manifestations were worrying about panicking or making a fool of oneself and increased heart rate without physical exertion; the least frequent being on the verge of panic and tremors. Lastly, in relation to stress symptoms, the most common were being unable to relax and having a hard time winding down, while the least frequent were being intolerant to obstacles to getting things done and expending a lot of nervous energy. Current major depression was diagnosed in 12.9% of the study participants. This figure is higher than that found in all previous studies with university students of both sexes [26,27,28,29,30], and in female university students [31,32].

The analysis of the variables predictive of depression showed that higher scores for life engagement were a protective factor, while higher scores in neuroticism and openness to experience were risk factors for depression. Previous research has demonstrated that greater life engagement constitutes a protective factor against depression in different segments of the population, which suggests that this may constitute an important psychological resource against stress [66,67], with stress levels being related to levels of psychological well-being [68]. Although previous research on life engagement as a predictor of depression in the university population is scarce, our findings are consistent with the work of Rossi et al. [43] among Chilean university students but differ from the findings of Liu et al. [42] with Japanese students, as Liu et al. did not find that life engagement predicted depression in their sample.

In relation to personality variables, university students with higher levels of neuroticism had a higher prevalence of depression. This finding is consistent with previous research [45,46,47,48]. However, there is little existing research on greater openness to experience as a predictor of depression among female university students, though our results are consistent with the work of Aldridge and Gore [45] with American college students. Participants with high openness to experience often demonstrate increased awareness and receptivity to their feelings, thoughts, and impulses; a need for variety; a recurring need to magnify and examine experiences; and a greater tendency to experience their emotions intensely [69]. According to Depression Self-Awareness Theory [70], experiences of loss or failure cause participants to focus on the discrepancy between their actual and desired state, which in turn leads to negative affect and depression. Individuals with a high openness to experience may be more likely to experience a large discrepancy between their actual and desired states, which could expose them to a higher risk of depression.

The limitations of the present study include the fact that the diagnosis of major depression was made through an unstructured clinical interview. Although this interview was conducted by clinicians who were experts in the topic of study, based on diagnostic criteria from the DSM-5 [50], future research should use structured diagnostic tools such as the SCID-5-CV [71]. In addition, the cross-sectional nature of the present study prevents us from inferring causal relationships. Furthermore, this research was conducted at a single university in Spain, which could limit the generalizability of these results; however, the sample’s sociodemographic and academic profile is similar to that found for Spanish university women in general [3,62], which suggests that its findings may be generalizable to the rest of Spanish university women.

This study also has notable strengths. It is an important contribution to knowledge about the symptoms of depression, anxiety, and stress, the current prevalence of depression, and predictors of depression in university women internationally, and particularly for Spanish university women. It used a large, random sample that was stratified by subject area and level of studies, with a high response rate. It used a diagnostic tool administered by experienced and trained clinicians to make the diagnosis of current major depression. It provides valuable information on the most common symptoms of depression, anxiety, and stress in female university students, and on predictors (especially those of a clinical nature) that are extremely useful in detecting mental health problems and the development of interventions to alleviate them.

The results of the current study have important implications for clinical practice and research. The high level of depressive and anxiety symptoms found in this sample, together with the presence of a good number of students in the severe/very severe range for stress, highlight the need to implement measures for the detection, prevention, and intervention of depression and the promotion of mental health targeting female university students. In fact, there are already psychological interventions that have been shown to have positive (albeit small) effect sizes on depression, anxiety, and stress when administered in an online format [72], and large effect sizes when they were targeted at university students diagnosed with depression [73]. The finding in the current study that depressive symptomatology was higher for students aged 20 or younger, without a partner; that depressive and anxiety symptoms were higher for undergraduate students; and that depressive, anxious and stress symptomatology were higher for non-heterosexual college students, provides valuable information on the subgroups of female students with higher needs for mental health services. Identification of the most frequent symptoms, as well as clinical variables that constitute potential protective factors (e.g., life engagement) or risk factors (e.g., neuroticism and openness to experience) for major depression, are of great clinical utility for the design of interventions adapted to the specific needs of this population. Future research should consider the diagnosis of major depression using structured tools and longitudinal designs that allow causal relationships to be established between the predictors analyzed and the presence of major depressive disorder.

## 5. Conclusions

This study’s findings provide information of great relevance for the adoption of comprehensive measures to address mental health issues for female university students. It is one of the few methodologically rigorous studies to have been conducted anywhere in the world, and more particularly in Spain, with female university students. Its results reveal the high level of depression and anxiety symptoms that female university students experience, as well as the existence of a high percentage of female students who experience severe/very severe levels of stress and the alarming percentage of participants with major depression. These findings highlight the need to adopt measures that address mental health, especially major depression, in female university students. Identifying the most frequent symptoms and finding a series of increased-risk and protective factors adds evidence to the existing scientific literature and allows efforts to be directed towards those experiences that are most frequent in female university students. It also aids in the design of targeted interventions intended to modify the most significant clinical predictors.

## Figures and Tables

**Table 1:** Sociodemographic, academic, and clinical characteristics of the participants.

Variables	*n* = 871	%
Age		
≤20	490	56.3
>21	381	43.7
Housing situation		
Other	427	49.0
Friends	444	51.0
Financially independent		
No	802	92.1
Yes	69	7.9
Residence		
Rural	388	44.5
Urban	483	55.5
Family income		
1999 Euros or less	409	47.0
2000 or more	462	53.0
Relationship status		
Single	489	56.1
Partnered	382	43.9
Sexual orientation		
Other orientations	234	26.9
Heterosexual	637	73.1
Academic major		
Other	546	62.7
Social or legal sciences	325	37.3
Undergraduate or graduate degree		
Undergraduate	745	85.5
Graduate	126	14.5

**Table 2:** Participants’ levels and scores for depression, anxiety, and stress.

	Depression		Anxiety		Stress	
Scores						
M	5.6		4.5		6.9	
SD	5.3		4.4		4.8	
Range	0–21		0–20		0–21	
Levels	*n*	%	*n*	%	*n*	%
Normal	452	51.9	454	52.1	510	58.6
Mild	115	13.2	72	8.3	110	12.6
Moderate	147	16.8	146	16.8	133	15.3
Severe	65	7.5	76	8.7	83	9.5
Very severe	92	10.6	123	14.1	35	4.0

**Table 3:** Results of multivariate logistic regression analyses for predictors of major depression.

Variables	OR (95% CI)	*p*
Family income		
1999 Euros or less	1.20 (0.77–1.88)	0.413
2000 or more	1 [Reference]	
Relationship status		
Single	0.64 (0.41–1.02)	0.064
Partnered	1 [Reference]	
Sexual orientation		
Other orientations	1.05 (0.65–1.71)	0.846
Heterosexual	1 [Reference]	
Optimism	0.96 (0.90–1.01)	0.137
Social support	0.89 (0.72–1.11)	0.307
Life engagement	0.92 (0.87–0.98)	0.009
Extraversion	1.02 (0.96–1.08)	0.499
Agreeableness	1.01 (0.96–1.06)	0.800
Conscientiousness	1.01 (0.96–1.06)	0.692
Neuroticism	1.20 (1.12–1.28)	<0.001
Openness to experience	1.08 (1.02–1.14)	0.008

## Data Availability

The data that support the reported results can be requested from the Office of Gender Equality (OIX) of Santiago de Compostela University; oix@usc.es.

## Appendix A

**Table A1:** Depression, anxiety and stress scores according to the sociodemographic and academic variables.

	Depression			Anxiety			Stress		
Variables	M (SD)	t	p	M (SD)	t	p	M (SD)	t	p
Age									
≤20	6.1 (5.2)	3.170	0.002	4.7 (4.5)	1.466	0.143	7.0 (4.8)	0.612	0.541
>21	5.0 (5.0)			4.3 (4.4)			6.8 (5.0)		
Housing situation									
Other	5.7 (5.5)	0.525	0.600	4.6 (4.4)	0.320	0.749	6.9 (4.8)	0.270	0.787
Friends	5.5 (5.0)			4.5 (4.5)			6.8 (4.9)		
Financially independent									
No	5.7 (5.3)	1.815	0.070	4.6 (4.5)	0.794	0.427	6.9 (4.9)	0.646	0.518
Yes	4.5 (4.7)			4.1 (4.1)			6.5 (4.8)		
Residence									
Rural	5.8 (5.3)	0.791	0.429	4.3 (4.4)	−1.134	0.257	6.9 (4.8)	0.005	0.996
Urban	5.5 (5.2)			4.7 (4.5)			6.9 (4.9)		
Family income									
1999 Euros or less	5.9 (5.4)	1.315	0.189	4.7 (4.5)	1.109	0.268	7.1 (4.9)	1.524	0.128
2000 or more	5.4 (5.1)			4.4 (4.3)			6.6 (4.8)		
Relationship status									
Single	6.1 (5.4)	2.838	0.005	4.7 (4.4)	1.505	0.133	6.9 (4.7)	−0.064	0.949
Partnered	5.1 (5.0)			4.3 (4.4)			6.9 (5.0)		
Sexual orientation									
Other orientations	6.8 (5.7)	3.663	<0.001	5.5 (4.6)	4.141	<0.001	7.6 (5.1)	2.593	0.010
Heterosexual	5.2 (5.1)			4.1 (4.3)			6.6 (4.8)		
Academic major									
Other	5.7 (5.2)	−0.447	0.655	4.6 (4.4)	−0.668	0.505	7.1 (4.7)	−1.098	0.273
Social or legal sciences	5.6 (5.3)			4.4 (4.5)			6.7 (4.9)		
Undergraduate or graduate degree									
Undergraduate	5.9 (5.4)	3.667	<0.001	4.6 (4.5)	1.978	0.048	6.9 (4.9)	0.944	0.345
Graduate	4.2 (4.5)			3.8 (4.1)			6.5 (4.9)		

**Table A2:** Depression levels according to the sociodemographic and academic variables.

	Normal	Mild	Moderate	Severe	Very Severe	Chi-Square	p
	n (%)	n (%)	n (%)	n (%)	n (%)		
Depression							
Variables							
Age							
<20	235 (48.0)	69 (14.1)	86 (17.6)	41 (8.4)	59 (12.0)	7.845	0.097
>21	217 (57.0)	46 (12.1)	61 (16.0)	24 (6.3)	33 (8.7)		
Housing situation							
Other	230 (53.9)	51 (11.9)	64 (15.0)	33 (7.7)	49 (11.5)	4.143	0.387
Friends	222 (50.0)	64 (14.4)	83 (18.7)	32 (7.2)	43 (9.7)		
Financially independent							
No	408 (50.9)	110 (13.7)	136 (17.0)	60 (7.5)	88 (11.0)	5.704	0.222
Yes	44 (63.8)	5 (7.2)	11 (15.9)	5 (7.2)	4 (5.8)		
Residence							
Rural	195 (50.3)	56 (14.4)	61 (15.7)	29 (7.5)	47 (12.1)	3.309	0.507
Urban	257 (53.2)	59 (12.2)	86 (17.8)	36 (7.5)	45 (9.3)		
Family income							
1999 Euros or less	208 (50.9)	49 (12.0)	76 (18.6)	29 (7.1)	47 (11.5)	3.134	0.536
2000 or more	244 (52.8)	66 (14.3)	71 (15.4)	36 (7.8)	45 (9.7)		
Relationship status							
Single	237 (48.5)	64 (13.1)	89 (18.2)	39 (8.0)	60 (12.3)	7.163	0.128
Partnered	215 (56.3)	51 (13.4)	58 (15.2)	26 (6.8)	32 (8.4)		
Sexual orientation							
Other orientations	105 (44.9)	26 (11.1)	47 (20.1)	20 (8.5)	36 (15.4)	13.600	0.009
Heterosexual	347 (54.5)	89 (14.0)	100 (15.7)	45 (7.1)	56 (8.8)		
Academic major							
Other	282 (51.6)	75 (13.7)	99 (18.1)	33 (6.0)	57 (10.4)	5.665	0.226
Social or legal sciences	170 (52.3)	40 (12.3)	48 (14.8)	32 (9.8)	35 (10.8)		
Undergraduate or graduate degree							
Undergraduate	374 (50.2)	100 (13.4)	126 (16.9)	59 (7.9)	86 (11.5)	9.169	0.057
Graduate	78 (61.9)	15 (11.9)	21 (16.7)	6 (4.8)	6 (4.8)		

**Table A3:** Anxiety levels according to the sociodemographic and academic variables.

	Normal	Mild	Moderate	Severe	Very Severe	Chi-Square	p
	n (%)	n (%)	n (%)	n (%)	n (%)		
Anxiety							
Variables							
Age							
<20	246 (50.2)	42 (8.6)	81 (16.5)	49 (10.0)	72 (14.7)	3.299	0.509
>21	208 (54.6)	30 (7.9)	65 (17.1)	27 (7.1)	51 (13.4)		
Housing situation							
Other	224 (52.5)	32 (7.5)	73 (17.1)	39 (9.1)	59 (13.8)	0.893	0.926
Friends	230 (51.8)	40 (9.0)	73 (16.4)	37 (8.3)	64 (14.4)		
Financially independent							
No	416 (51.9)	67 (8.4)	136 (17.0)	67 (8.4)	116 (14.5)	2.890	0.576
Yes	38 (55.1)	5 (7.2)	10 (14.5)	9 (13.0)	7 (10.1)		
Residence							
Rural	215 (55.4)	24 (6.2)	59 (15.2)	39 (10.1)	51 (13.1)	8.010	0.091
Urban	239 (49.5)	48 (9.9)	87 (18.0)	37 (7.7)	72 (14.9)		
Family income							
1999 Euros or less	216 (52.8)	33 (8.1)	58 (14.2)	36 (8.8)	66 (16.1)	5.394	0.249
2000 or more	238 (51.5)	39 (8.4)	88 (19.0)	40 (8.7)	57 (12.3)		
Relationship status							
Single	245 (50.1)	44 (9.0)	82 (16.8)	43 (8.8)	75 (15.3)	2.769	0.597
Partnered	209 (54.7)	28 (7.3)	64 (16.8)	33 (8.6)	48 (12.6)		
Sexual orientation							
Other orientations	98 (41.9)	20 (8.5)	43 (18.4)	32 (13.7)	41 (17.5)	18.571	0.001
Heterosexual	356 (55.9)	52 (8.2)	103 (16.2)	44 (6.9)	82 (12.9)		
Academic major							
Other	295 (54.0)	41 (7.5)	87 (15.9)	46 (8.4)	77 (14.1)	2.785	0.594
Social or legal sciences	159 (48.9)	31 (9.5)	59 (18.2)	30 ().2)	46 (14.2)		
Undergraduate or graduate degree							
Undergraduate	378 (50.7)	63 (8.5)	129 (17.3)	66 (8.9)	109 (14.6)	4.113	0.391
Graduate	76 (60.3)	9 (7.1)	17 (13.5)	10 (7.9)	14 (11.1)		

**Table A4:** Stress levels according to the sociodemographic and academic variables.

	Normal	Mild	Moderate	Severe	Very Severe	Chi-Square	p
	n (%)	n (%)	n (%)	n (%)	n (%)		
Stress							
Variables							
Age							
<20	281 (57.3)	67 (13.7)	79 (16.1)	45 (9.2)	18 (3.7)	2.251	0.690
>21	229 (60.1)	43 (11.3)	54 (14.2)	38 (10.0)	17 (4.5)		
Housing situation							
Other	250 (58.5)	52 (12.2)	70 (16.4)	42 (10.1)	12 (2.8)	4.127	0.389
Friends	260 (58.6)	58 (13.1)	63 (14.2)	40 (9.0)	23 (5.2)		
Financially independent							
No	467 (58.2)	103 (12.8)	125 (15.6)	74 (9.2)	33 (4.1)	2.416	0.660
Yes	43 (62.3)	7 (10.1)	8 (11.6)	9 (13.0)	2 (2.9)		
Residence							
Rural	234 (60.3)	44 (11.3)	58 (14.9)	37 (9.5)	15 (3.9)	1.377	0.848
Urban	275 (57.1)	66 (13.7)	75 (15.5)	46 (9.5)	20 (4.1)		
Family income							
1999 Euros or less	233 (57.0)	49 (12.0)	67 (16.4)	43 (10.5)	17 (4.2)	2.032	0.730
2000 or more	277 (60.0)	61 (13.2)	66 (14.3)	40 (8.7)	18 (3.9)		
Relationship status							
Single	283 (57.9)	67 (13.7)	78 (16.0)	46 (9.4)	15 (3.1)	3.968	0.410
Partnered	227 (59.4)	43 (11.3)	55 (14.4)	37 (9.7)	20 (5.2)		
Sexual orientation							
Other orientations	128 (54.7)	24 (10.3)	42 (17.9)	27 (11.5)	13 (5.6)	6.978	0.137
Heterosexual	382 (60.0)	86 (13.5)	91 (14.3)	56 (8.8)	22 (3.5)		
Academic major							
Other	328 (60.1)	68 (12.5)	77 (14.1)	51 (9.3)	22 (4.0)	1.973	0.741
Social or legal sciences	182 (56.0)	42 (12.9)	56 (17.2)	32 (9.8)	13 (4.0)		
Undergraduate or graduate degree							
Undergraduate	432 (58.0)	96 (12.9)	117 (15.7)	68 (9.1)	32 (4.3)	3.044	0.550
Graduate	78 (61.9)	14 (11.1)	16 (12.7)	15 (11.9)	3 (2.4)		

**Table A5:** Distribution of responses to the depression, anxiety, and stress items.

	Not at All Applicable to Me		Applicable Some of the Time		Applicable a Good Part of the Time		Applicable Most of the Time	
	n	%	n	%	n	%	n	%
Depressive subscale								
3. Not feeling positive emotions	505	58.0	237	27.2	96	11.0	33	3.8
5. Difficult working up initiative	314	36.1	297	34.1	172	19.7	88	10.1
10. Nothing to look forward to	451	51.8	206	23.7	125	14.4	89	10.2
13. Down-hearted and blue	230	26.4	318	36.5	197	22.60	126	14.5
16. No enthusiasm for anything	560	64.3	176	20.2	89	10.2	46	5.3
17. Feeling that one has no worth as a person	469	53.8	194	22.3	113	13.0	95	10.9
21. Life is meaningless	612	70.3	128	14.7	64	7.3	67	7.7
Anxiety subscale								
2. Dry mouth	436	50.1	268	30.8	121	13.9	46	5.3
4. Difficulty breathing	556	63.8	182	20.9	97	11.1	36	4.1
7. Tremors	587	67.4	155	17.8	93	10.7	36	4.1
9. Worrying about panicking or making a fool of oneself	426	48.9	221	25.4	125	14.4	99	11.4
15. Bordering on panic	626	71.9	147	16.9	72	8.3	26	3.0
19. Increased heart rate without physical exertion	473	54.3	209	24.0	111	12.7	78	9.0
20. Being scared without good reason	530	60.8	177	20.3	113	13.0	51	5.9
Stress subscale								
1. Hard to wind down	255	29.3	294	33.8	195	22.4	127	14.6
6. Overreaction to situations	352	40.4	257	29.5	180	20.7	82	9.4
8. Using a lot of nervous energy	422	48.5	238	27.3	148	17.0	63	7.2
11. Feeling agitated	359	41.1	272	31.2	178	20.4	62	7.1
12. Unable to relax	246	28.2	259	29.7	236	27.1	130	14.9
14. Intolerant of obstacles to getting things done	540	62.0	198	22.7	100	11.5	33	3.8
18. Feeling touchy	337	38.7	265	30.4	171	19.6	98	11.3
